# Supplementary material for: Protein-mediated RNA folding governs sequence-specific interactions between rotavirus genome segments
Source: eLife. 2017 Sep 18;6:e27453. doi: 10.7554/eLife.27453 (PMC5621836; doi:10.7554/eLife.27453)
Supplement: Supplementary file 1. — RNA samples were measured at 25°C in 10 mM HEPES-Na, pH 7.4, 150 mM NaCl, 0.5 mM MgCl2. For each RNA sample, photon data were acquired and 20 autocorrelation functions (ACFs) were calculated, as described in Materials and methods. The ACF data were fitted to a one-component diffusion model with dark state dynamics (Materials and methods) to calculate their respective diffusion time values for each RNA sample, and their corresponding diffusion coefficients and hydrodynamic radii were computed assuming the diffusion coefficient of the free ATTO488 dye (D = 400 μm2/s at 25°C), as described in Materials and methods. Diffusion coefficients and hydrodynamic radii are reported as mean ±SD for at least 3 RNA samples preparations. [file elife-27453-supp1.docx]

**Supplementary Table 1.** Estimated diffusion coefficients (D) and hydrodynamic radii (R_h_) of the fluorescently labelled RV segment precursors ssRNAs, measured by FCS at 25^o^C in 10 mM HEPES-Na, pH 7.4, 150 mM NaCl, 0.5 mM MgCl_2_

| **RNA** | **Length, nt** | **D, μm^2^/s** | **R*_h_*, nm** |
| --- | --- | --- | --- |
| **S1** | 3302 | 12±2.1 | 17.3±3 |
| **S2** | 2687 | 12.2±0.7 | 17.0±1 |
| **S3** | 2591 | 12.9±1 | 16.1±1.2 |
| **S4** | 2362 | 12.8±1.4 | 16.3±1.8 |
| **S5** | 1578 | 15.5±1.1 | 13.9±1 |
| **S6** | 1356 | 14.4±1 | 14.4±1 |
| **S7** | 1063 | 22±2 | 9.5±0.9 |
| **S8** | 1059 | 19±1.2 | 11±0.7 |
| **S9** | 1074 | 19±1 | 11±0.7 |
| **S10** | 751 | 24 ±1.2 | 8.7± 0.5 |
| **S11** | 667 | 23.2±4.3 | 7.4±1.4 |
| **S11 & S10, incubated with ssRNAs S1 to S9 in the presence of BSA** | | | |
| **S11** | 667 | 19±3 | 10.9±1.7 |
| **S10** | 751 | 22.78±2 | 9.1±0.8 |

For each RNA sample, photon data were acquired and 20 autocorrelation functions (ACFs) were calculated, as described in Materials and Methods. The ACF data were fitted to a one-component diffusion model with dark state dynamics (Materials and Methods) to calculate their respective diffusion time values for each RNA sample, and their corresponding diffusion coefficients and hydrodynamic radii were computed assuming the diffusion coefficient of the free ATTO488 dye (D = 400 μm^2^/s at 25^o^C), as described in Methods. Diffusion coefficients and hydrodynamic radii are reported as mean±SD for at least 3 RNA samples preparations.
