# Supplementary material for: Protein-mediated RNA folding governs sequence-specific interactions between rotavirus genome segments
Source: eLife. 2017 Sep 18;6:e27453. doi: 10.7554/eLife.27453 (PMC5621836; doi:10.7554/eLife.27453)
Supplement: Supplementary file 2. — DNA oligonucleotides used for site-directed mutagenesis and cloning purposes and RNA oligonucleotides used for FCS measurements. Genbank accession numbers are given for genome segment cDNA templates used for the in vitro transcription of S1-S11 and Non-RV control RNAs. All RNA and DNA oligonucleotides and gBlock DNA sequences were obtained commercially from Integrated DNA Technologies. [file elife-27453-supp2.docx]

**Supplementary File 2. DNA and RNA sequences used in the study**

| S5mut_R | atatcttcctCTGCTAACCATGTATGAAAC  agaagaaaacTTACTACATCTTTAAATGTTCGC |
| --- | --- |
| S5mut_F |  |
| S11mut_5_F | agatcatatatgAAAAATGAATCATCTTCAACAACG |
| S11mut_5_R | tctttatcttcctAGACTCGTCACGTCAATAC |
| S11mut_s3_493_F | tataaaGACGATTCTGATAGTGATG |
| S11mut_s3_493_R | aattttATCTGAATCAGATTCTGC |
| S6mut_1124_F | aagctaACTAACTATTCACCATCTAGAG |
| S6mut_1124_R | aagttagCAATTCATACCTGGTGGAAAAAC |
| S6mut_1243_F | atacataatctGTCAAGCTGTTTGAACTC |
| S6mut_1243_R | aagttatctataGGATACCAAGTGGTTAGC |
| S11mut_s3_566_F | tattatactattaAATGCAATTGATCGAAGATTTG |
| S11mut_s3_566_R | tgttataaattttTGCGAAATACTTCCTCTTATATTTAC |
| S11_comp_5F | ATAGAAGAAGTATATACAAAAATGAATCATCTTC |
| S11_comp_5R | ATCTTCCTAGACTCGTCACGTCAATAC |
| ‘Scrambled’ RNA DNA template gBlock | TAAGCTT*TAATACGACTCACTATAGG*CCTTTTGCAGCGCTCCGG  TGACCTCCCGCGCAAGCGAGGTTTGGAGTCATGCGTCTCTGTCGTCGACT  ATATACAAGAGTGGATCATCTGCGGCCACGTCAACCCGGCTACGATTCACT  CAGGCTAGAGGGGATCAATAGCTCTGAGTCGATACAGCACAATTCCCTGA  ATGCAAACCGTACAAGTCTCGGCGAGAAATGAGACCAGCAGATTGTACTT  GCAACGAATCACGGACCATCTATACGGAGAGATCGAAAGGCGTTCAGCCT  ACTACCTACCATCGCCTGTTAATCGACTCTTAAACGATCAAAGGGTCATCGG  TGAACGTACGGACCTATTGGCGGGGTTTGTCCCTTACGAAAGGAAGTAGT  ACGAAAGCGAACCTAGGTTCATGTATACCCCTATCAGGCAGTTCGAGTAGA  GGAAAGGCCTAGGGCGACGATAAATCAAGGGGACTCTAGCCGGAAGAGG  AAGTGGCATCGGATGAAGTGGACAAGACGCTCGAGGGTGCTGAGAGTGG  TGATGATTAACGTAAATATTGTAAAGATAATACGAAGCCTTCCGAAAAAAGA  ACCAGAATGAAACAGTGTGCAAGACAAGGGATCGAAGATTTGGGTCCCAA  ACCTATGAGGGCACTCGAGAGCCCGACACTCCCCTTGAGTGACGGGATCC |
| F_pet28_NcoI_NSP2 | ATATATCCATGGCTGAGCTAGCTTGCTTTTGTTA |
| R_pet28_XhoI_NSP2 | ATATATCTCGAGTGGAATTCCTATTTGAGAGACCT |
| RNA SELEX template | ATGACGCCAAGGCAGGTCTAT(N30)TGATACCAGCTCCAACGCGTC,  N30 – any nucleotide |
| SELEX P1 | GACGCGTTGGAGCTGGTATCA |
| SELEX P2 | AGTAATACGACTCACTATAGGGAAATGACGCCAAGGCAGGTCTAT |
| Seq11_84-100 RNA | 5’-Cy3-AUC-AUC-UUC-AAC-AAC-GU-3’ |
| DNA templates for *in vitro* transcription of S1-S11 RNAs | GenBank IDs: S1: KF729687.1; S2: KF729639.1; S3: KF729647.1; S4: KF729690.1; S5: KF729657.1; S6: KF729692.1; S7: KF729693.1; S8: KF729694.1; S9: KF729695; S10: KF729696.1; S11: KF729697.1 |

*All RNA and DNA oligonucleotides and gBlock DNA sequences were obtained commercially from Integrated DNA Technologies (IDT).
