# Supplementary figures and images for: Protein-mediated RNA folding governs sequence-specific interactions between rotavirus genome segments
Source: eLife. 2017 Sep 18;6:e27453. doi: 10.7554/eLife.27453 (PMC5621836; doi:10.7554/eLife.27453)

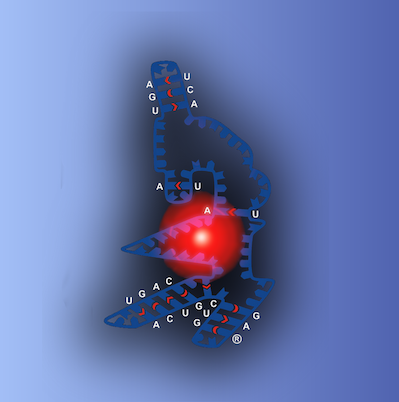

Supplement: Source code 1. [file elife-27453-code1.zip › images/avatar.png]

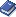

Supplement: Source code 1. [file elife-27453-code1.zip › images/BurstBrowser/book_sim.jpg]

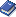

Supplement: Source code 1. [file elife-27453-code1.zip › images/BurstBrowser/book_sim.png]

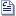

Supplement: Source code 1. [file elife-27453-code1.zip › images/BurstBrowser/cc_sourcecodec.jpg]

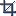

Supplement: Source code 1. [file elife-27453-code1.zip › images/BurstBrowser/crop_tool.jpg]

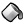

Supplement: Source code 1. [file elife-27453-code1.zip › images/BurstBrowser/Floodfill_24.jpg]

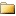

Supplement: Source code 1. [file elife-27453-code1.zip › images/BurstBrowser/folder.jpg]

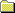

Supplement: Source code 1. [file elife-27453-code1.zip › images/BurstBrowser/foldericon.gif]

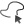

Supplement: Source code 1. [file elife-27453-code1.zip › images/BurstBrowser/Freehand_24px.jpg]

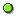

Supplement: Source code 1. [file elife-27453-code1.zip › images/BurstBrowser/greencircleicon.gif]

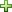

Supplement: Source code 1. [file elife-27453-code1.zip › images/BurstBrowser/GreenPlus_12.jpg]

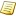

Supplement: Source code 1. [file elife-27453-code1.zip › images/BurstBrowser/help_rn.jpg]

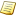

Supplement: Source code 1. [file elife-27453-code1.zip › images/BurstBrowser/help_rn.png]

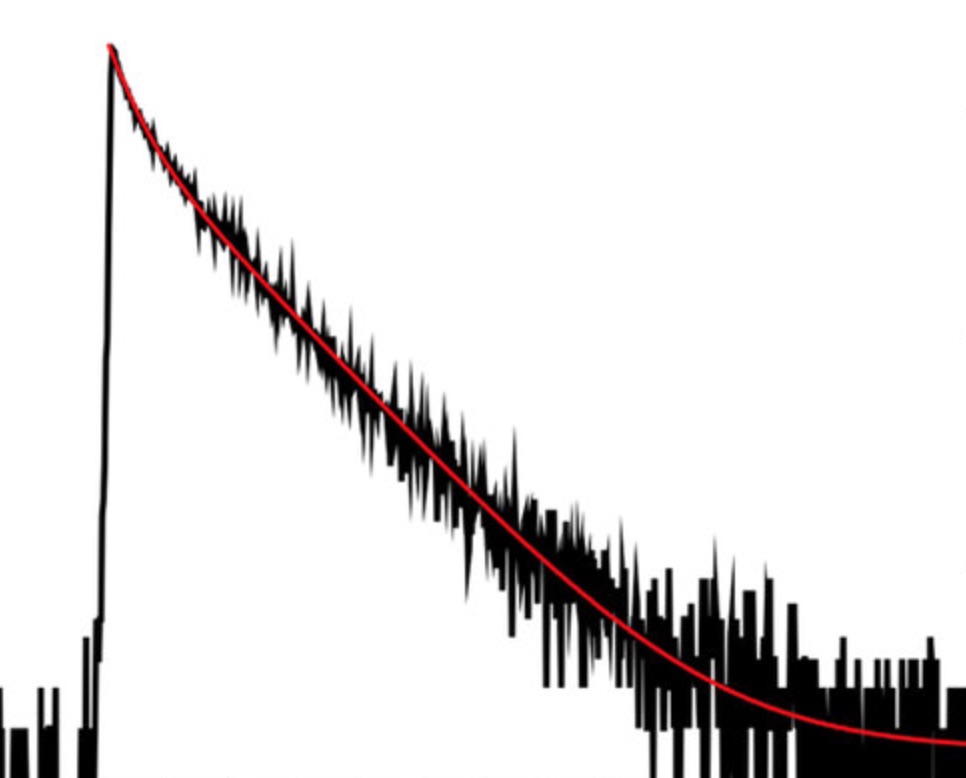

Supplement: Source code 1. [file elife-27453-code1.zip › images/BurstBrowser/lifetime.jpg]

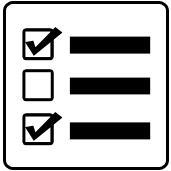

Supplement: Source code 1. [file elife-27453-code1.zip › images/BurstBrowser/multiselection.png]

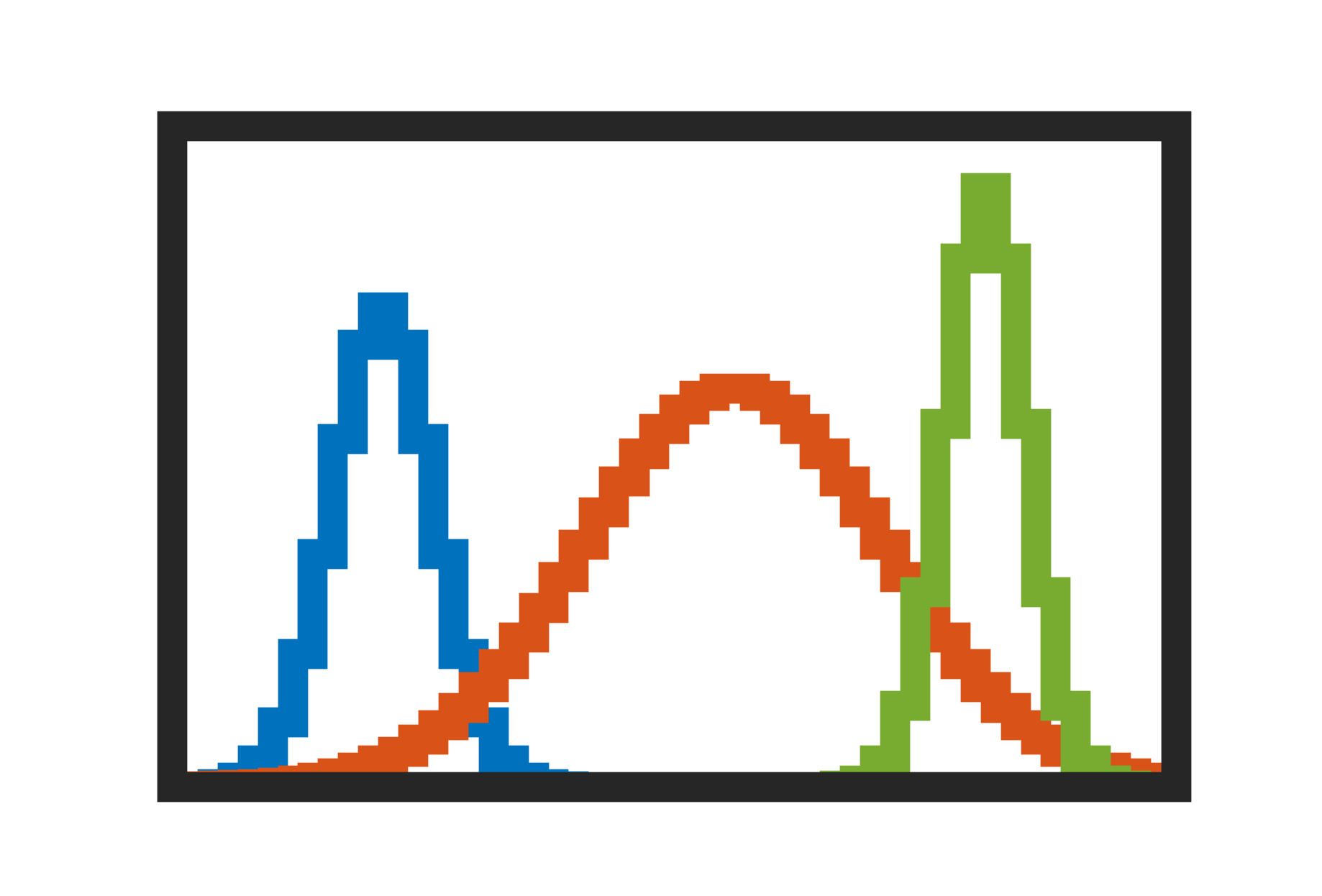

Supplement: Source code 1. [file elife-27453-code1.zip › images/BurstBrowser/plot_multiple_icon.png]

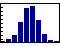

Supplement: Source code 1. [file elife-27453-code1.zip › images/BurstBrowser/plottype-hist.gif]

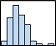

Supplement: Source code 1. [file elife-27453-code1.zip › images/BurstBrowser/plottype-hist.jpg]

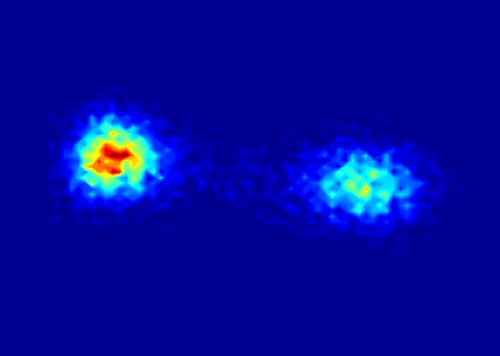

Supplement: Source code 1. [file elife-27453-code1.zip › images/BurstBrowser/splash.jpg]

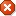

Supplement: Source code 1. [file elife-27453-code1.zip › images/BurstBrowser/status_failed.jpg]

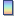

Supplement: Source code 1. [file elife-27453-code1.zip › images/BurstBrowser/tool_colorbar.png]

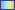

Supplement: Source code 1. [file elife-27453-code1.zip › images/BurstBrowser/zscale.png]

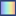

Supplement: Source code 1. [file elife-27453-code1.zip › images/BurstBrowser/zscale_square.png]

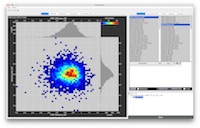

Supplement: Source code 1. [file elife-27453-code1.zip › images/Launcher/BurstBrowser.jpg]

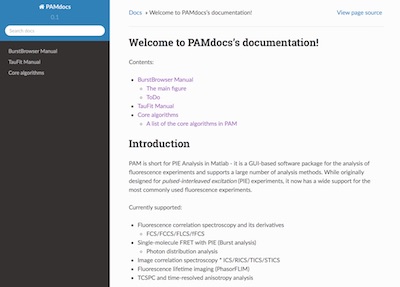

Supplement: Source code 1. [file elife-27453-code1.zip › images/Launcher/Doc.jpg]

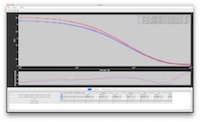

Supplement: Source code 1. [file elife-27453-code1.zip › images/Launcher/FCSFit.jpg]

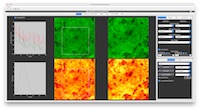

Supplement: Source code 1. [file elife-27453-code1.zip › images/Launcher/MIA.jpg]

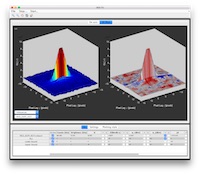

Supplement: Source code 1. [file elife-27453-code1.zip › images/Launcher/MIAFit.jpg]

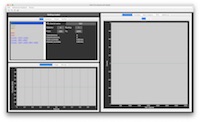

Supplement: Source code 1. [file elife-27453-code1.zip › images/Launcher/Pam.jpg]

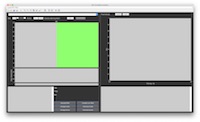

Supplement: Source code 1. [file elife-27453-code1.zip › images/Launcher/PCF.jpg]

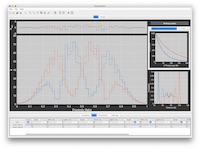

Supplement: Source code 1. [file elife-27453-code1.zip › images/Launcher/PDA.jpg]

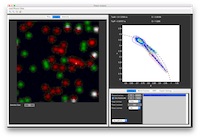

Supplement: Source code 1. [file elife-27453-code1.zip › images/Launcher/Phasor.jpg]

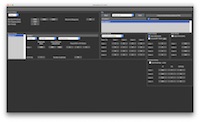

Supplement: Source code 1. [file elife-27453-code1.zip › images/Launcher/Sim.jpg]

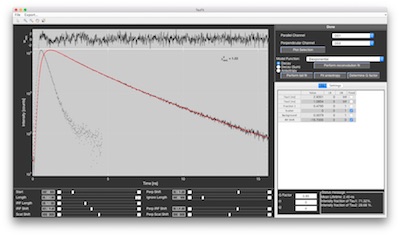

Supplement: Source code 1. [file elife-27453-code1.zip › images/Launcher/TauFit.jpg]

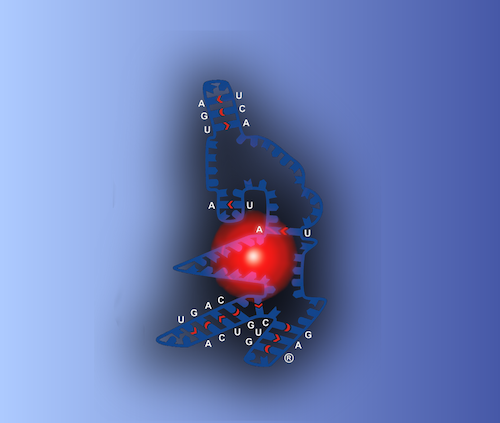

Supplement: Source code 1. [file elife-27453-code1.zip › images/PAM/logo.png]

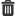

Supplement: Source code 1. [file elife-27453-code1.zip › images/trash16p.png]
